# Adding a smartphone app to global postural re-education to improve neck pain, posture, quality of life, and endurance in people with nonspecific neck pain: a randomized controlled trial

**DOI:** 10.1186/s13063-021-05214-8

**Published:** 2021-04-12

**Authors:** Fatemeh Abadiyan, Malihe Hadadnezhad, Zohre Khosrokiani, Amir Letafatkar, Haniyeh Akhshik

**Affiliations:** 1 Faculty of Physical Education and Sport Sciences, Department of Biomechanics and Sport injuries, Kharazmi University, Tehran, Iran; 2 Biomechanics and Corrective Exercise Laboratory, Faculty of Physical Education and Sport Sciences, Kharazmi University, Mirdamad Blvd., Hesari St, Tehran, 00982122258084 Iran

**Keywords:** Neck pain, Posture, Musculoskeletal pain, Musculoskeletal abnormalities, Disability

## Abstract

**Background:**

In this study, the effect of adding a smartphone app to an 8-week global postural reeducation (GPR) on neck pain, endurance, quality of life, and forward head posture (FHP) in patients with chronic neck pain and FHP was evaluated.

**Methods:**

Sixty male and female office workers (38.5 ± 9.1 years) with chronic neck pain were randomly assigned into three groups: group 1 (GPR+ a smartphone app, *n* = 20), group 2 (GPR alone, *n* = 20), and group 3 (the control group, *n* = 20). The primary outcome was pain and the secondary outcomes were disability, quality of life, endurance, and posture. Pain, disability, endurance, quality of life, and posture were evaluated using the visual analog scale (VAS), neck disability index (NDI), progressive iso-inertial lifting evaluation (PILE) test, quality of life questionnaire (SF-36), and photogrammetry, respectively, at pre-and post-8-week interventions. A one-way analysis of covariance (ANCOVA) has been conducted to statistically analyze the data.

**Results:**

The GPR+ a smartphone app had statistically significant improvements versus GPR alone in pain (mean difference, − 2.05 ± 0.65, ES (95% CI) − 0.50 (− 1.04 to − 0.01), *P* = 0.04), disability (difference = 11.5 ± 1.2, ES (95% CI) = 0.31 (0.22 to 0.97), *p* = 0.033), FHP (difference = 1.6 ± 0.2, ES (95% CI) = 0.31 (0.09 to 0.92), *p* = 0.047), and endurance (difference = 2 ± 3.3, ES (95% CI) = 0.51 (0.02 to 1.03), *p* = 0.039). Both of the GPR+ a smartphone app and GPR alone groups had statistically significant differences versus the control group in all outcomes.

**Conclusion:**

When a workplace assessment and management could not be as part of any intervention, adding a smartphone app to GPR for NP may be an appropriate tool to administer a home and work exercise program resulting in elevating pain and disability, as well as improving FHP and endurance.

**Trial registration:**

Current Controlled Trials using the UMIN-RCT website UMIN000039720. Retrospectively registered on January 9, 2020.

**Supplementary Information:**

The online version contains supplementary material available at 10.1186/s13063-021-05214-8.

## Introduction

Non-specific is a common musculoskeletal complaint [1] with an incidence of 42–63% in computer office workers [2]. When treatment comes to patients with neck pain (NP), changes relative to pain, function, and disability must be considered as important symptoms [3]. Moreover, it has been shown that computer users complain about the decrease ability to control head posture and mobility because of reduced sense and abnormal proprioception in their neck joints [4]. Environmental (prolonged static or awkward postures, highly repetitive movements and computer work) and physical (inadequate strength or muscle endurance and poor posture) factors contribute to the development of work-related neck pain [5]. A recent systematic review recommended level II evidence for strengthening exercise to relieve pain, but it has reported that the effect of endurance and stretching exercise has to be more investigated [6]. However, some authors proposed that NP can be managed through different exercise programs [7, 8] such as global postural reeducation (GPR) [9–12].

GPR is an alternative conservative treatment to manage NP [10–12]. This therapeutic strategy focuses on stretching the postural muscles organized as “muscle chains,” of which two are anterior and posterior chains [11]. Specifically, GPR focuses on stretching the shortened muscles and facilitating the activity of the antagonists’ muscles by using prolonged active postures to improve the muscle balance and postural symmetry [13]. There remains a need for further studies to investigate how to enhance the effect of a therapeutic exercise treatment on computer users complaining of musculoskeletal complaints [4].

Moreover, the large spread of smartphone technology and its software applications, coupled with the popularity of mobile technologies, now leads to take smartphones as a tool to help patients and the health care system of the future, based on self-management of a home exercise program. Smartphones are easy to use, relatively inexpensive, and highly accessible [14]. With the use of apps that can be downloaded onto the smartphone, a patient could perform a program according to the prepared schedule and completely independent to the healthcare system [15]. For Iranian users, Isfahani et al. suggested that a smartphone app can be used to administer a patient’s program [15]. The use of such apps increases patient’s awareness regarding the time, type, and dose of the exercises and decreases the misunderstanding about the program [16]. So, it may enhance accuracy during functional tasks, increase patients’ engagement in their rehabilitation and postural control, and reduce the need for ongoing contacts with the healthcare professionals to monitor implementing rehabilitation programs [16].

A recent systematic review (2018) investigating on effective exercise in preventing a new episode of neck pain reported high-quality randomized, controlled trials are needed to evaluate effectiveness of an exercise intervention alone without health information/stress management training and a workplace assessment as part of the intervention [7]. To the knowledge of the authors, there is no randomized controlled trial (RCT) adding a smartphone app to GPR for NP, to administer a patient’s home exercise program without a workplace assessment as part of the intervention.

Therefore, this research aimed to compare the effect of GPR with and without a smartphone app on the pain, disability, endurance, forward head posture (FHP), and quality of life in patients with NP and FHP. It was hypothesized that adding a smartphone app would enhance treatment effects on pain, disability, endurance, FHP, and quality of life in people with NP and FHP.

## Material and methods

### Study design

This RCT (registered at UMIN-RCT, ID number, UMIN000039720) with blind assessor was carried out in the Kharazmi University. All data collection was performed in the laboratory of the university. Prior to the enrollment, all subjects were informed about the objectives of the study and provided written informed consent. This study was performed in accordance with the 1964 Helsinki declaration, its later amendments and local ethics committee, and approved by Kharrazmi University Institutional Review Board in human subjects (DBSI12052019).

### Participants and randomization

Participants, including male and female office workers with chronic neck pain, were recruited by physical therapists through flyers displayed at physical therapy clinics and hospitals between September 2018 and January 2019, in Tehran, Iran. Of 100 participants enrolled for the study, 60 met the inclusion criteria were randomly assigned into group 1 (GPR + a smartphone app, *n* = 20), group 2 (GPR, *n* = 20), and the control group (*n* = 20). An independent researcher applied randomization by using computer-generated numbers, which were stratified based on age and sex to avoid clustering across study groups.

Participants were randomized by the slot-drawing method to GPR alone, GPR + a smartphone app, or control groups based on a blocked randomization. The randomization sequence was not disclosed until participants had completed their baseline assessments. The allocation was by sealed opaque envelopes. This study was conducted as a pre-post intervention with blinded assessors.

Inclusion criteria for this study were office workers (using a computer at least 4 h), aged between 28 and 48 years, NP between 3 and 8 cm on a visual analog scale (VAS) (from 0 indicating no pain at all to 10 indicating unbearable pain), chronic nonspecific neck pain lasting for more than 3 months, and FHP less than 46° [17]. On the other side, exclusion criteria included specific causes of NP (e.g., systemic, rheumatic, neuromuscular diseases), central or peripheral neurological signs, cognitive impairment, spinal surgery, or physical therapy treatments in the last 6 months prior to the baseline assessment [11, 17]. Participants with NP were allowed to take part in the study according to the inclusion and exclusion criteria by an experienced physiotherapist.

Participants were asked not to receive any extra intervention for NP. Participants would be also excluded if they missed at least three consecutive or four non-consecutive sessions.

### Procedure

All participants were provided with a neck pain brochure, containing some practical instructions and pictures to correct their postures during the different daily activities [11], and they were methodically informed of the study details. The characteristics of all participants were recorded through an employee profile, including age, gender, job titles, employment status, hours worked with a computer per day (at least 4 h/day), and type of work performed.

The participants were assessed by an experienced physiotherapist based on clinical history, posture, and symptom responses to active movements. In addition, the participants were screened by measuring the craniovertebral angle (CVA less than 46°) with photogrammetry to determine FHP [18]. CVA has good intra-rater reliability (ICC ≥ 0.85) [18]. CVA was measured as the angle between an imaginary line extended from C7 through the tragus, and the horizontal line.

### Outcome measures

Pain during the last 24 h was the primary outcome, while disability, endurance, FHP, and quality of life were secondary outcomes. All variables measured two times in each group at the baseline and after 8-week interventions. Outcome measures were administered each time by an assessor blinded to the participants’ allocated groups. Also, the therapists and participants were blinded to other groups.

#### Neck pain

NP was evaluated using the 10-cm VAS. This scale is widely used in clinical settings to assess the effectiveness of pain treatment (ICC = 0.81) [19]. The participants indicated their current pain level by choosing a number from 0 (no pain at all) to 10 (unbearable pain) displayed along a horizontal line [17, 20]. The minimum clinically important difference for within-group on the pain scale has been reported 2.5 points in people with NP as a baseline score indicated greater than 6.0 based on patient satisfaction after treatment [21].

#### Quality of life

The quality of life questionnaire (SF36) was used to evaluate the patients’ life quality with a mental score (the intraclass correlation coefficient (ICC) = 0.68), SF-36 physical score (ICC = 0.65) [22]. The SF-36 has been also used to test the correlation between health-related quality of life and related factors (sex, age, physical function, and daily functioning) rated on a 5-point scale [23]. It consists of 36 items and 8 scales, including physical function, physical role, body pain, general health, vitality, social behavior, emotional role, and mental health. The obtained scores range between 0 and 100 with higher scores indicating better health status and a mean score of 50 as a normative value [22, 24]. The minimum clinically important difference for quality of life is 10 points for NP [21].

#### Assessment of FHP

To assess FHP, CVA is reported to have good intra-rater reliability with ICC ≥ 0.85 [18]. Furthermore, CVA is the angle between an imaginary line from C_7_ to the tragus and the horizontal line [17, 25]. CVA is the position of the head relative to the trunk. The smaller CVA is, the greater FHP will be [25]. The participants were asked to sit on a seat; markers were placed over the right tragus, acromion process, and C_7_ spinous process. A digital camera (Canon SX720 HS) was also placed on a tripod 1 m high and 3.5 m away from the wall. FHP was measured using image processing software (kinovea.0.8.15) by the respective angles between the centers of the markers and the horizontal line. The normative value for FHP was an angle less than 46° [17]. The minimum clinically important difference for the measurement of FHP angle is 3.31 or more based on patient satisfaction after treatment [26].

#### Endurance

Endurance was measured by the progressive isoinertial lifting evaluation (PILE) test. PILE test has been recommended as a functional test to measure muscle endurance (ICC ≥ 0.85) [27]. Moreover, PILE involved lifting weights from waist to shoulder height (30–54 in.) for people with neck pain. Participants began with an 8-pound load and a 13-pound load for females and males respectively. Weight was subsequently increased at a rate equal to the initial free weight every 20 s. Four lifting movements were actually performed at 20-s intervals. The test end-point was also established when aerobic capacity or neuromuscular fatigue being felt. Gender differences were considered for neck endurance assessment [17, 20]. To realize unbiased conditions, a blind assessor evaluated PILE. To be experienced in the evaluation of the test, the assessor had a 3-day training according to PILE. As for musculoskeletal pain, the minimum clinically important difference scale has been reported 8.5 IB to 15.5 IB [28].

#### Neck disability index (NDI)

Disability was evaluated using a neck disability index (NDI) questionnaire. The NDI is 10 questions and each question has a possible total score of zero to 5. The total score from all 10 questions is divided by 50, the maximum score possible, with total scores expressed in percentage. Higher percentage scores indicate a worse disability. The NDI has good to excellent internal consistency and moderate to excellent reliability and poor to good responsiveness. The MCID has been reported 20% in a similar cohort of people with chronic neck-related symptoms [29].

### Interventions

Following randomization, the groups 1 and 2 underwent the experimental interventions about 50 min a day, 4 days a week for an 8-week period. Group 1, also, performed exercises which were reminded by a smartphone app at predetermined times. The participants in the control group performed an evidence-based physiotherapy program about 50 min a day, 4 days a week for an 8-week period, described as a postural correction on daily activities. The participants in each experimental group performed the intervention in a clinic and were supervised by physiotherapists and two corrective exercise trainers specialized at postural reeducation exercises by the physiotherapists. Progression of the exercises was prescheduled but flexible according to each individual’s progression and limitation.

To maximize the adherence to the treatment allocations, besides explaining the importance of the exercise intervention to the participants at the initial of each session, the participants were informed how the program would positively affect their symptoms and daily activities by the strong research team. Participants in GRP alone and control groups were not informed that there was another group who had a smartphone app to alarm about their correct postures and home exercises.

#### Global postural reeducation

The GPR involves a series of active gentle movements and postures aimed at realignment of the joints, stretching shortened muscles, and enhancing the contraction of antagonist’s muscles. Indeed, the GPR includes eight therapeutic postures, including lying, sitting, or standing, each held for 15/20 min. To reduce variability between sessions and trainers, only 2 postures among the 8 proposed ones were used. GPR was employed and progressed in a tailored way for each participant [30].

Exercise 1, the lying posture with leg extension progression, was aimed to stretch the anterior muscle chain (diaphragm, pectoralis minor, scalene, sternocleidomastoid, intercostalis, iliopsoas, arm, forearm, and hand flexors) [30]. To perform such a posture, the participant lied down while hips flexed, abducted, externally rotated, and palms of the feet together with the upper limb in supination with about 30° of abduction. Progression in the posture was to extend the lower limbs and adduct the upper ones while maintaining the soles of the feet together, in alignment with the body axis (exercise 1).

Exercise 2, the lying posture with flexion of the thighs, was intended to stretch the posterior chain (upper trapezius, levator scapulae, suboccipital, erector spinae, gluteus maximus, ischiotibial, triceps surae, and foot intrinsic muscles). Hence, the initial position was lying with the hip flexed, and progression consisted of increasing hip flexion, knee extension, and dorsiflexion of the ankle while maintaining the soles of the feet together, in aligned with the body axis (exercise 2) [30].

In order to complete the research exercise procedure, each posture was practiced for about 15 min. During each GPR session, besides manual traction on both cervical and lumbar area, based on the participants’ postures and tolerance, the physiotherapists instructed the participants how to contract and hold then release their shortened muscles. In optimizing the stretching and discouraging compensatory movements during the postural exercise, each participant was encouraged to be in the right alignment and make the necessary correction while verbal commands and manual contact were applied by the physiotherapist. In each of the postural exercise, the physical therapist advanced the posture to the limit for each volunteer. Each GPR session was followed by performing neck movements while the whole spinal segments and pelvic were in a position taught by PTs. The physiotherapists trained the participants to integrate the postural correction in their daily activities according to their capabilities. All participants in the intervention groups were asked to perform written recommendations for their daily activities taught at the first session by the physiotherapists at home [11]. The recommendations explained how to carry a weight, how to work on a work station, how to sit on a sofa, how to sit while reading a book or using a tablet, how to reach an object and to do activities close to the ground, and how to sleep [11].

#### A smartphone app

In this study, an app called “Seeb” (Android Studio software) was used, which can be easily installed on a smartphone. “Seeb” was installed for the participants’ smartphones at the predetermined times (an interval of 300 s for correcting posture described in the treatment sessions and twice in days they did not undergo GPR, for performing exercises to correct their daily activities) based on self-managed of work time and a home exercise program. The developed application was installed on Android operating system versions 4–6. The smartphone made a beep sound followed by showing the picture of the exercises and correct postures during daily activities [15]. The goal was to decrease potential risk factors for musculoskeletal disorders by the reminders [31].

“Seeb” could store the name and descriptions of a self-manage exercise program, type and the repetition of each exercise, insertion of the exercise picture, the record of administration instruction, the record of the user reaction to the warning (exercise administration or non-administration), and the exercise administration schedule (the time of the first and last exercise performing, the repeat hours of performing, and exercise refill reminder) [15].

#### The control group intervention

Participants in the control group (*n* = 20) received evidence-based physiotherapy, including “traditional” neck education and exercise therapy, focused on issues such as anatomy, physiology and biomechanics of the spine, common causes of spinal (neck) pain, the load-tolerance model, nociceptive pain processing, the importance of self-care, and ergonomic suggestions about daily activities including standing, sitting, and lifting [3].

### Statistical analysis

The SPSS software was used to statistically analyze the data (IBM Corp., Armonk, NY, USA).

The necessary sample size was estimated using G*Power 3.1.7 for Windows (G*Power©, University of Dusseldorf, Germany). The sample size calculation was considered a power calculation to detect between-group differences in the primary outcome measure (neck pain). To obtain 80% statistical power (1-β error probability) with an α error level probability of 0.05, we used repeated-measure analysis of variance (ANOVA), within-between interaction, and a medium effect size to consider two groups and two measurements for the primary outcome, generating a sample size about of 17 participants per group (total sample size of 51 subjects). The sample was increased to 60 (20 in each group) to allow for a 15% dropout rate. A total of 60 subjects met study criteria and participated in the study [17].

One-way analysis of variance (ANOVA) was used to compare the group demographics, and post hoc independent *t* tests were performed in the case of a significant omnibus test. The dependent variables of interest were pain, endurance, FHP angle, and quality of life. For each variable, the 3-trial mean was calculated for each patient. One-way analysis of covariance (ANCOVA), with a between-factor of a group (GPR+ a smartphone app, GPR alone or control groups) and participant baseline scores included as a covariate, was used to determine if there were group differences in the dependent variables of interest at post-testing. For each variable, the percentage of change was calculated compared with baseline.

This analysis approach (i.e., post-test performance as the outcome with baseline performance as a covariate) allowed us to compare post-testing outcomes while accounting for potential baseline group differences [32]. In the case of a significant omnibus test, pairwise comparisons were performed to examine potential between-group differences. These pairwise comparisons were based on the adjusted group means. In addition, 95% confidence intervals (95% CI) were calculated based on the adjusted group mean differences, and Cohen’s *d* effect size (ES) statistics were calculated by dividing the adjusted group mean differences by the larger of the group standard deviations. The Bryant-Paulson procedure was used when conducting the pairwise comparisons and calculating the confidence intervals [33]. An alpha of 0.05 was used for all significance tests [34]. Effect sizes of 0.2, 0.5, and 0.8 were considered “small,” “moderate,” and “large,” respectively [34].

## Results

No statistically significant differences were observed between the GPR+ a smartphone app, GPR alone, or control groups as with participants’ age, mass, computer using time, pain duration, and sex, and no difference was observed with mass between the 2 data collection time points (*P* > .05 Table 1).

**Table 1:** Participants demographics and characteristics

	GPR+ AF	GPR alone	Control	***P*** value
**Age, years; mean (SD)**	41.3 (8.1)	40.3 (7.9)	37.4 (9.8)	0.09
**Weight, kg; mean (SD)**	63.5 (6.6)	62.2 (7.6)	59.8 (6.1)	0.13
**Gender—male/female;** ***n*** **(%)**	10/10 (50)	10/10 (50)	7/13 (35)	0.09
**Pain, VAS**	7.3 ± 0.9	6.7 ± 1.2	6.4 ± 1.8	0.22
**Duration of symptoms;** ***n*** **(%)**				
3–12 months	7 (35)	9 (45)	6 (30)	0.39
13–36 months	12 (60)	10 (50)	12 (60)	0.34
> 36 months	1 (5)	1 (5)	2 (10)	0.42
**Computer (h/day)**	5	5	5	1.00

*GPR group* global postural reeducation group, *GPR + AF group* global postural reeducation + acoustic feedback group

**Fig. 1:**
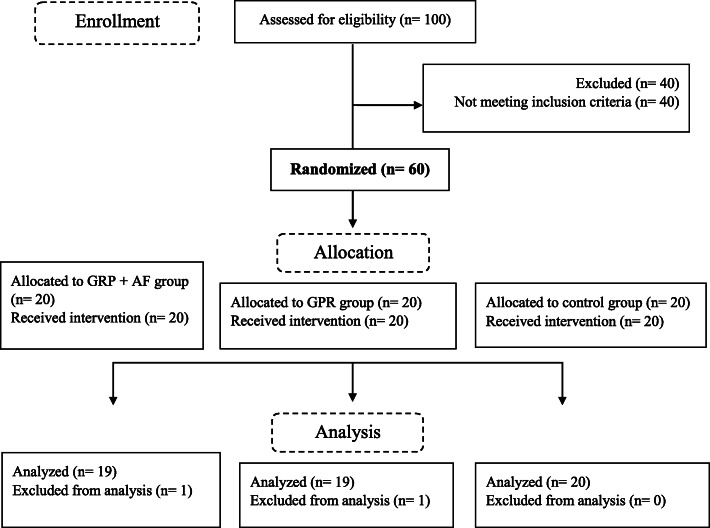
Flow diagram of the study

There were 2 participants decided to leave the study because of personal reasons (1 for GPR+ acoustic feedback, and 1 for GPR alone). Hence, the number of all participants decreased to 58 (97%) (Fig. 1). Also, there was a high degree of adherence to all three interventions. Of the possible 24 sessions, participants attended 21 ± 2 in GPR+ a smartphone app, 21 ± 2 in the GPR alone, and 22 ± 1 in the control groups. No adverse event was reported.

The group × time interaction is presented in Table 2. The group who received GPR+ a smartphone app indicated more improvement as compared to the one who received GPR alone. Significant main time effects were found for pain (*F* = 7.45, *P* = 0.03), disability (*F* = 24.33, *P* < 0.001), FHP (*F* = 18.83, *P* = 0.02), endurance (*F* = 14.65, *P* = 0.03), and quality of life (*F* = 11.67, *P* = 0.03). Moreover, no significant main time effects were found for RMDQ.

**Table 2:** Within and between-group changes in the outcomes (pain, disability, FHP, endurance, and QoL)

		Mean ± SD		Within-group			Between-groups	
Variable	Groups	Baseline	8 weeks	Change relative to baseline^§^ (%)	Effect size^†^ and 95% confidence interval (lower limit–upper limit)	*p*	Interaction effects	*P*
**Pain, VAS**	GPR+ AF	7.3 ± 0.9	4.4 ± 1.7	39.7% ↓	0.89 (0.34 to 1.64)	0.01^‡^	*F* = 7.45 *P* = 0.03^Ω^	0.031* 0.041^Ф^
	GPR alone	6.7 ± 1.2	5.8 ± 1.05	13.4% ↓	0.23 (0.26 to 1.22)	0.06		
	Control	6.4 ± 1.8	6.9 ± 1.6	9.3% ↑	− 0.06 (− 0.04 to 0.38)	0.45		
**Disability, degree**	GPR+ AF	36.3 ± 6.1	19.3 ± 6	46.8% ↓	0.98 (0.6 to 2.45)	0.01^‡^	*F* = 24.33 *P* = 0.01^Ω^	0.001* 0.031^¥^ 0.037^Ф^
	GPR alone	34 ± 6.6	28.5 ± 5.3	16.2% ↓	0.56 (0.38 to 1.27)	0.04^‡^		
	Control	37 ± 7.4	39.3 ± 6.4	2.5% ↑	− 0.04 (− 0.01 to 0.55)	0.41		
**FHP, degree**	GPR+ AF	38.5 ± 4.3	42.1 ± 3.6	9.3% ↑	− 0.68 (− 1.9 to − 0.22)	0.02^‡^	*F* = 18.83 *P* = 0.02^Ω^	0.001* 0.021^¥^ 0.047^Ф^
	GPR alone	38.9 ± 3.3	40.9 ± 2.4	5.1% ↑	− 0.56 (− 1.64 to − 0.39)	0.03^‡^		
	Control	39.8 ± 2.4	39.4 ± 2.7	1% ↓	− 0.09 (− 0.06 to 1.01)	0.36		
**Endurance, scale**	GPR+ AF	49.5 ± 12.8	60.2 ± 17.6	21.6% ↑	− 0.75 (− 1.27 to − 0.11)	0.01^‡^	*F* = 14.65 *P* = 0.03^Ω^	0.001* 0.001^¥^ 0.039^Ф^
	GPR alone	58 ± 16.8	66.7 ± 15.3	15% ↑	− 0.46 (− 0.91 to − 0.04)	0.02^‡^		
	Control	46.9 ± 10.5	46.3 ± 9.3	1.3% ↓	0.04 (− 0.06 to 1.01)	0.67		
**Quality of life, scale**	GPR+ AF	69.1 ± 4.0	78.5 ± 3.6	13.6% ↑	− 0.77 (− 1.9 to − 0.22)	0.03^‡^	*F* = 11.67 *P* = 0.03^Ω^	0.001* 0.009^¥^
	GPR alone	69.2 ± 4.5	77.5 ± 5.5	12% ↑	− 0.76 (− 1.64 to − 0.39)	0.03^‡^		
	Control	69.8 ± 6	71.6 ± 7.7	2.6% ↑	− 0.11 (− 0.09 to 0.68)	0.53		

*Abbreviations*: *GPR group* global postural reeducation group, *GPR + AF group* global postural reeducation + acoustic feedback group, *VAS* visual analog scale, *FHP* forward head posture

*Significant between combined global postural reeducation with acoustic feedback and control groups

^¥^Significant between global postural reeducation alone and control groups

^Ф^Significant between combined global postural reeducation with acoustic feedback and global postural reeducation alone groups

^§^Percent change (↓ decrease, ↑ increase)

^‡^Significant within group improvement between the baseline and 8-week treatment period

^Ω^Significant group × time interaction

### Primary outcome measure

For pain, at 8 weeks, the GPR+ a smartphone app group (ES = 0.89, *p* = 0.01) had significant within-group changes, but differences in the GPR alone (ES = 0.23, *p* = 0.06) and control (ES = − 0.06, *p* = 0.45) groups were not significant. Differences between GPR+ a smartphone app vs. control (difference = 3 ± 0.6, ES (95% CI) = − 0.77 (− 1.29 to − 0.24), *p* = 0.031) and GPR+ a smartphone app vs. GPR alone (difference = − 2.05 ± 0.65, ES (95% CI) = − 0.50 (− 1.04 to − 0.01), *p* = 0.041) were significant (Table 2).

### Secondary outcome measure

For disability, at 8 weeks, both of the GPR+ a smartphone app (ES = 0.98, *p* = 0.01) and GPR alone (ES = 0.56, *p* = 0.04) groups had significant within-group changes, but differences in the control group (ES = − 0.04, *p* = 0.41) was not significant. Differences between GPR+ a smartphone app vs. control (difference = 19.3 ± 0.9, ES (95% CI) = 1.45 (0.65 to 1.62), *p* = 0.001), GPR alone vs. control (difference = 7.8 ± 0.3, ES (95% CI) = 0.43 (0.08 to 1.02), *p* = 0.027), and GPR+ a smartphone app vs. GPR alone (difference = 11.5 ± 1.2, ES (95% CI) = 0.31 (0.22 to 0.97), *p* = 0.033) groups were significant (Table 2).

For FHP, at 8 weeks, both of the GPR+ a smartphone app (ES = − 0.68, *p* = 0.02) and GPR alone (ES = − 0.56, *p* = 0.03) groups had significant within-group changes, but differences in the control group (ES = − 0.09, *p* = 0.36) were not significant. Differences between GPR+ a smartphone app vs. control (difference = 4 ± 0.4, ES (95% CI) = 1.03 (0.49 to 1.57), *p* = 0.001), GPR alone vs. control (difference = 2.4 ± 0.6, ES (95% CI) = 0.61 (0.10 to 1.13), *p* = 0.021), and GPR+ a smartphone app vs. GPR alone (difference = 1.6 ± 0.2, ES (95% CI) = 0.31 (0.09 to 0.92), *p* = 0.047) were significant (Table 2).

For endurance, at 8 weeks, both of the GPR+ a smartphone app (ES = − 0.75, *p* = 0.01) and GPR alone (ES = − 0.46, *p* = 0.02) groups had significant within-group changes, but differences in the control group (ES = 0.04, *p* = 0.67) were not significant. Differences between GPR+ a smartphone app vs. control (difference = 11.3 ± 3.6, ES (95% CI) = 1.23 (0.9 to 1.64), *p* = 0.001), GPR alone vs. control (difference = 9.3 ± 0.3, ES (95% CI) = 1.12 (0.73 to 1.76), *p* = 0.001), and GPR+ a smartphone app vs. GPR alone (difference = 2 ± 3.3, ES (95% CI) = 0.51 (0.02 to 1.03), *p* = 0.039) were significant (Table 2).

For quality of life, at 8 weeks, both of the GPR+ a smartphone app (ES = − 0.77, *p* = 0.03) and GPR alone (ES = − 0.76, *p* = 0.03) groups had significant within-group changes, but differences in the control group (ES = − 0.11, *p* = 0.53) were not significant. Differences between GPR+ a smartphone app vs. control (difference = 7.6 ± 1.2, ES (95% CI) = 1.96 (1.34 to 2.57), *p* = 0.001) and GPR alone vs. control (difference = 6.5 ± 0.7, ES (95% CI) = 1.67 (1.08 to 2.26), *p* = 0.009) were significant (Table 2).

## Discussion

To our knowledge, this is the first study to investigate the effects of a GPR + a smartphone app to improve symptoms of people with NP and FHP. The results showed that GPR+ a smartphone app lead to a greater relieving in NP, and improvements in disability, endurance and FHP, but not in the quality of life among people with NP as compared with GPR alone.

Using a smartphone app dramatically could reduce the time caring for a patient and the rate of medicine errors [16]. Even small errors in any stage of treatment may have a large detrimental impact on the processes of the treatment. When the task becomes familiar to a person, it can reduce disruptive anxiety and cognitive workload and the risk of errors [35].

Moreover, the research shows that even motivated people may forget to perform the exercise [36]. A smartphone has the potential to use not only as the simplest possible solution, i.e., a timer-based alert, but also a more sophisticated technology. This could be possible if take into account users’ behavior and the unique nature of their daily routines [36].

The receptor systems of human body may provide ambiguous or incorrect information during normal activities to control posture [37]. To compensate irrelevant information during any abnormal posture, the body may require ability to adapt and control the correct posture by means of a reminder as a feedback [38].

Feedback is reported to have significant effects on improving pain, disability, and surface electromyography of the selected muscles as compared to active and passive interventions on managing work-related neck and shoulder pain [39]. Medians et al. suggested that feedback may augment rehabilitation of the upper limb in the chronic phase such as following stroke [40]. Also, education and augmented feedback about correct posture could be implemented to treat computer users with FHP while working with a computer [4]. Alerting about a sustained posture could decrease the effect of unstandardized ergonomics during working with computers. The alerting could be through a mirror or a file [41].

Also, GPR challenges the whole kinetic chain of the people with NP which demonstrate the abnormal activation between superficial and deep muscles and co-activation of agonist and antagonist muscles. GPR actively engages a patient in eliciting and maintaining an improved postural alignment; such active engagement presumably added an element of motor learning that could enhance behavioral change [11]. Additionally, enhancing motor learning using signals could improve posture and muscle activation in people with NP and FHP. These signals alert the users to their posture or work hours in a determined time [31]. The reminding about a posture as extrinsic feedback could be applied to users with neck pain to improve their abnormal sensory (or intrinsic) feedback which could provide incorrect information about posture [42].

Pillastrini et al. [13] demonstrated that GPR was more effective as compared to manual therapy to improve pain and disability at the post-treatment and a 6-month follow-up in patients with chronic nonspecific neck pain [13]. Moreover, Amorim et al. (2013) reported that GPR was significantly associated with improvements in function, pain, and the quality of life [10]. Having conducted GPR intervention, in another study, Radhakrishnan et al. [43] reported significant improvement in quality of life [43].

Therefore, improvement in the examined variables could be resulted from prolonged stretching and increased self-perception and postural awareness during the 8-week GPR [10, 12]. Also, the outcomes received more improvement using the app reminder to correct the posture while showing pictures which may be considered as augmented signals alerting about incorrect posture and abnormal intrinsic information during the sustained posture [42].

As with most studies, this study has few limitations which may make difficult to interpret the effects of GPR+ a smartphone app on other variables. As no group had a mean neck angle > 46° after the interventions, there is a need for a more duration intervention. Unfortunately, changes in the activations of neck and shoulder muscles and the neck range of motion were not evaluated, while they are of high importance in the treatment of neck pain. Investigating the effect of GPR+ smartphone app on the neck range of motion, and neck and shoulder muscle activations are recommended for the future studies with large sample size on people with NP with FHP. Another limitation is it is not clear if the adherence to the program could be implemented to move from passive alerts to a smarter memory and routine assistant [36] or just a smartphone addiction in a long time. So, large sample, high-quality, adequately powered, randomized controlled trials are required to follow up the results.

## Conclusion

The results of this study may be implemented in the clinical settings, using economical intervention and equipment. Our results implied that the GPR+ a smartphone app showed better relieving in pain and improvements in endurance and FHP as compared with the GPR alone and education in male and female workers with nonspecific neck pain. When a workplace assessment and management could not be as part of any intervention, adding a smartphone app to GPR for NP may be an appropriate tool to administer a home and work exercise program resulting in elevating pain and disability, as well as improving FHP and endurance.

## Supplementary Information

**Additional file 1.** Research protocol.

**Additional file 2.** RCT Protocol.

**Additional file 3.** Exercise 1- Start.

**Additional file 4.** Exercise 1-End.

**Additional file 5.** Exercise 2-Start.

**Additional file 6.** Exercise 2-End.

## Data Availability

The datasets analyzed during the current study are available from the corresponding author on reasonable request.

## Abbreviations

NP: Neck pain
GPR: Global postural reeducation
FHP: Forward head posture
VAS: Visual analog scale
PILE: Progressive iso-inertial lifting evaluation
SF-36: Quality of life questionnaire
ANOVA: Analysis of variance
CVA: Craniovertebral angle
