# Editorial Expression of Concern: Adding a smartphone app to global postural re-education to improve neck pain, posture, quality of life, and endurance in people with nonspecific neck pain: a randomized controlled trial

**DOI:** 10.1186/s13063-026-09450-8

**Published:** 2026-01-14

**Authors:** Fatemeh Abadiyan, Malihe Hadadnezhad, Zohre Khosrokiani, Amir Letafatkar, Haniyeh Akhshik

**Affiliations:** 1 Faculty of Physical Education and Sport Sciences, Department of Biomechanics and Sport Injuries, Kharazmi University, Tehran, Iran; 2 Biomechanics and Corrective Exercise Laboratory, Faculty of Physical Education and Sport Sciences, Kharazmi University, Mirdamad Blvd., Hesari St, Tehran, 00982122258084 Iran

**Editorial Expression of Concern: Trials 22, 274 (2021)**

**https://doi.org/10.1186/s13063-021-05214-8**

The Editor-in-Chief is issuing an editorial expression of concern to alert readers that concerns have been raised regarding the ethical approval for this research. The authors have provided conflicting documentation regarding which Institutional Review Board approved their work, the approval numbers and the dates at which these approvals were received.

Amir Letafatkar does not agree to this editorial expression of concern. Fatemeh Abadiyan, Malihe Hadadnezhad, Zohre Khosrokiani and Haniyeh Akhshik have not responded to any correspondence from the editor about this editorial expression of concern.

The original article [1], can be found online at https://link.springer.com/article/10.1186/s13063-021-05214-8.
