# Supplementary material for: Adding a smartphone app to global postural re-education to improve neck pain, posture, quality of life, and endurance in people with nonspecific neck pain: a randomized controlled trial
Source: Trials. 2021 Apr 12;22:274. doi: 10.1186/s13063-021-05214-8 (PMC8042925; doi:10.1186/s13063-021-05214-8)
Supplement: Supplementary file 1 — Additional file 1. Research protocol. [file 13063_2021_5214_MOESM1_ESM.doc]

**Research protocol: part 1**

### Project summary

This study evaluated the effect of adding a smartphone App to an eight-week global postural reeducation (GPR) on neck pain, endurance, quality of life and forward head posture (FHP) in patients with chronic neck pain and FHP. Sixty male and female office workers (38.5±9.1 years) with chronic neck pain were randomly assigned into three groups: Group 1 (GPR+ a smartphone App, n=20), Group 2 (GPR alone, n=20) and Group 3 (the control group, n=20). The primary outcome was pain and the secondary outcomes were disability, quality of life, endurance and posture. All the subjects were evaluated using the visual analog scale (VAS), neck disability index (NDI), progressive iso-inertial lifting evaluation (PILE), quality of life questionnaire (SF-36) and photogrammetry at pre- and post- eight-week interventions. The GPR+ a smartphone App had statistically significant improvements versus GPR alone in pain (mean diﬀerence, -2.05±0.65, ES (95%CI): -0.50(-1.04 to -0.01), P= 0.04), disability (difference = 11.5±1.2, ES (95% CI) = 0.31 (0.22 to 0.97), p=0.033), FHP ((difference = 1.6±0.2, ES (95% CI) = 0.31 (0.09 to 0.92), p=0.047), and endurance (difference = 2±3.3, ES (95% CI) = 0.51 (0.02 to 1.03), p=0.039). Both of the GPR+ a smartphone App and GPR alone groups had statistically significant differences versus the control group in all outcomes. This study suggests that adding a smartphone app to GPR could significantly relieve pain, and improve disability, endurance, forward head angle and quality of life in people with neck pain and FHP compared with GPR alone. Further research is needed to confirm the clinically improvement differences on symptoms in people with neck pain and FHP when adding a smartphone app to GPR.

### General information

- Protocol title, protocol identifying number (if any), and date: **Adding a smartphone App to Global Postural Re-education to improve Neck Pain, Posture, Quality of Life, and Endurance in People with Nonspecific Neck Pain: A Randomized Controlled Trial**

This RCT (registered at [UMIN-RCT](https://fa.irct.ir/), registration number, UMIN000039720, registration date, 08/08/2018) with blind assessor carried out in the Kharazmi University.

- Name and address of the sponsor/funder: None.
- Name and title of the investigator(s) who is (are) responsible for conducting the research, and the address and telephone number(s) of the research site(s), including responsibilities of each

Malihe Hadadnezhad, PhD., Faculty of Physical Education and Sport sciences, Department of Biomechanics and Sport injuries, Kharazmi University, Tehran-Republic of Iran

Address: Biomechanics and Corrective Exercise Laboratory, Faculty of Physical Education and Sport sciences, Kharazmi University, Mirdamad Blvd., Hesari St, Tehran, Iran. Postal code: 00982122258084.

TEL: +989195394692 - +982634207184

E-mail Address: m[.hadadnezhad@yahoo.com](mailto:.hadadnezhad@yahoo.com)

A.L., Z.KH., M.H., H.A, and F.A. prepared study design and implement a testing, A.L., and Z.KH. wrote the main manuscript text and M.H. and Z.KH. participate in final edition of article.

- Name(s) and address(es) of the clinical laboratory(ies) and other medical and/or technical department(s) and/or institutions involved in the research.

| Kharazmi University |  |
| --- | --- |
| **Division name** | department of biomechanics and sport injuries |
| **Zip code** | 4352763633 |
| **Address** | tehran-mirdamad-sout razan street, faculty of PE |
| **TEL** | 009802122258084 |

### Rationale & background information

Moreover, the large spread of smartphone technology and its software applications, coupled with the popularity of mobile technologies, now leads to take smartphones as a tool to help patients and the health care system of the future, based on self-management of a home- exercise program. Smartphones are easy to use, relatively inexpensive, and highly accessible. With the use of apps that can be downloaded onto the smartphone, a patient program could be performed according to the prepared schedule and completely independent to the healthcare system. For Iranian users, Isfahani et al, (2017) suggested to use a smartphone app to administer a patient’s program. The use of such apps increases patient’s awareness regarding the time, type and dose of the exercise, and decreases the misunderstanding about the program. So, it may enhance accuracy during functional tasks, increase patients’ engagement in their rehabilitation and postural control, and reduce the need for ongoing contacts with the healthcare professionals to monitor implementing rehabilitation programs. To the knowledge of the authors, there is no randomized controlled trial (RCT) adding a smartphone app to GPR for NP, to administer a patient’s home- exercise program.

### References (of literature cited in preceding sections)

1. Ye S, Jing Q, Wei C, Lu J. Risk factors of non-specific neck pain and low back pain in computer-using office workers in China: a cross-sectional study. BMJ Open. 2017; 7(4): 1-7.
2. Chen X, Coombes BK, Sjogaard G, Jun D, O'Leary S, & Johnston V. Workplace-Based Interventions for Neck Pain in Office Workers: Systematic Review and Meta-Analysis. Phys Ther. 2018; 98(1): 40-62.
3. Bragatto MM, Bevilaqua-Grossi D, Regalo SC, Sousa JD, Chaves TC. Associations among temporomandibular disorders, chronic neck pain and neck pain disability in computer office workers: a pilot study. J Oral Rehabil. 2016; 43(5): 321-332.
4. Korthals-de Bos IB, Hoving JL, van Tulder MW, Rutten-van Mölken MP, Adèr HJ, de Vet HC, Koes BW, Vondeling H, Bouter LM. Cost effectiveness of physiotherapy, manual therapy, and general practitioner care for neck pain: economic evaluation alongside a randomised controlled trial. BMJ. 2003 Apr 26; 326(7395):911.
5. Blanpied PR, Gross AR, Elliott JM, et al. Neck Pain: Revision 2017. J Orthop Sports Phys Ther. 2017;47(7):1-83.
6. Kang JH, Park RY, Lee SJ, Kim JY, Yoon SR, Jung KI. The effect of the forward head posture on postural balance in long time computer based worker. Ann Rehabil Med. 2012; 36: 98-104.
7. Wang WTJ, Olson SL, Campbell AH, Hanten WP, Gleeson PB. Effectiveness of physical therapy for patients with neck pain: an individual approach using a clinical decision-making algorithm. Am J Phys Med Rehabil. 2003;82: 203-218.
8. de Campos TF, Maher CG, Steffens D, Fuller JT, Hancock MJ. Exercise programs may be effective in preventing a new episode of neck pain: a systematic review. Journal of Physiotherapy 2018; 64: 159-165
9. Shiravi S, Letafatkar A, Bertozzi L, Pillastrini P, Khaleghi Tazji M. Efficacy of Abdominal Control Feedback and Scapula Stabilization Exercises in Participants with Forward Head, Round Shoulder Postures and Neck Movement Impairment. Sports Health, 2019; 11(3): 272-279.
10. Lynch SS, Thigpen CA, Mihalik JP, Prentice WE, & Padua D. The effects of an exercise intervention on forward head and rounded shoulder postures in elite swimmers. Br J Sports Med. 2010; 44(5): 376-381.
11. Amorim CS, Gracitelli ME, Marques AP, & Alves VL. Effectiveness of global postural reeducation compared to segmental exercises on function, pain, and quality of life of patients with scapular dyskinesis associated with neck pain: a preliminary clinical trial. J Manipulative Physiol Ther. 2014; 37(6): 441-447.
12. Pillastrini P, Banchelli F, Guccione A, Di Ciaccio E, Violante FS, Brugnettini M, Vanti C. Global Postural Reeducation in patients with chronic nonspecific neck pain: cross-over analysis of a randomized controlled trial. Med Lav. 2018; 109(1): 16-30.
13. Cunha AC, Burke TN, Franca FJ, Marques AP. Effect of global posture reeducation and of static stretching on pain, range of motion, and quality of life in women with chronic neck pain: a randomized clinical trial. Clinics. 2008; 63(6): 763-770.
14. Pillastrini P, de Lima ESRF, Banchelli F, Burioli A, Di Ciaccio E, Guccione AA, Vanti C. Effectiveness of Global Postural Re-education in Patients with Chronic Nonspecific Neck Pain: Randomized Controlled Trial. Phys Ther. 2016; 96(9): 1408-1416.
15. Keogh JWL, Espinosa HG, Grigg J. Evolution of smart devices and human movement apps: recommendations for use in sports science education and practice. Journal of Fitness Research. 2016; 5:14-15.
16. Isfahani S, Ehteshami A, Savari E, Samimi A. Developing the Medication Reminder Mobile Application Seeb. Acta Informatica Medica, 2017; 25(2), 108. doi:10.5455/aim.2017.25.108-111 (<https://doi.org/10.5455/aim.2017.25.108-111>)
17. Siebert JN, Ehrler F, Combescure C, Lacroix L, Haddad K, Sanchez O. A Mobile Device App to Reduce Time to Drug Delivery and Medication Errors During Simulated Pediatric Cardiopulmonary Resuscitation: A Randomized Controlled Trial. J Med Internet Res. 2017; 19(2).

### Study goals and objectives

This research was aimed to compare the effect of GPR with and without a smartphone app on the pain, disability, endurance, FHP and quality of life in patients with neck pain and FHP. It was hypothesized that adding a smartphone app would enhance treatment effects on pain, disability, endurance, FHP and quality of life in people with neck pain and FHP.

### Study design

Participants, including male and female office workers with chronic neck pain, were recruited by physical therapists through flyers displayed at physical therapy clinics and hospitals between Sep 2018 and Jan 2019, in Tehran, Iran. Of 100 participants enrolled for the study, 60 met the inclusion criteria were randomly assigned into Group 1 (the GPR a smartphone app, n=20), Group 2 (the GPR, n=20) and the control group (n=20). An independent researcher applied randomization by using computer generated numbers, which were stratified based on age and sex to avoid clustering across study groups.

Participants were randomized by the slot-drawing method to GPR alone, GPR with a smartphone app or control groups. The randomization sequence was not disclosed until participants had completed their baseline assessments. Allocation was by sealed opaque envelopes. This study was conducted as a pre-post intervention with blinded assessors.

Inclusion criteria for this study were office workers (using a computer at least 4 hours), aged between 28 and 48 years, neck pain between 3-8 cm on a visual analog scale (VAS) (from 0 indicating no pain at all to 10 indicating unbearable pain), chronic nonspecific neck pain lasting for more than 3 months, and FHP less than 46°. On the other side, exclusion criteria included specific causes of neck pain (e.g. systemic, rheumatic, neuromuscular diseases), central or peripheral neurological signs, cognitive impairment, spinal surgery, or physical therapy treatments in the last 6 months prior to the baseline assessment. Participants with neck pain were allowed to take part in the study according to the inclusion and exclusion criteria by an experienced physiotherapist.

The subjects were asked not to receive any extra intervention for neck pain. Participants would be also excluded if they missed at least three consecutive or four nonconsecutive sessions.

### Methodology

All the participants were provided with a neck pain brochure, containing some practical instructions and pictures to correct their postures during the different daily activities,^12^ and they were methodically informed of the study details. The characteristics of all participants were recorded through an employee profile, including age, gender, job titles, employment status, hours worked with computer per day (at least 4 hours/day), and type of work performed.

The participants were assessed by an experienced physiotherapist based on clinical history, posture and symptom responses to active movements. In addition, the subjects were screened by measuring the craniovertebral angle (CVA less than 46°) with photogrammetry to determine FHP. The craniovertebral angle has a good intra-rater reliability (ICC ≥ 0.85). The CVA was measured as the angle between an imaginary line extended from C7 through the tragus, and the horizontal line.

### Safety considerations

No adverse event was reported.

### Follow-up

Not applicable.

### Data management and statistical analysis

One-way analysis of variance (ANOVA) was used to compare the group demographics and post hoc independent t-tests were performed in the case of a significant omnibus test. The dependent variables of interest were the pain, endurance, FHP angle, and quality of life. For each variable the 3-trial mean was calculated for each patient. One-way analysis of covariance (ANCOVA), with a between-factor of group (GPR+ a smartphone app, GPR alone or control groups) and participants baseline scores included as a covariate, was used to determine if there were group differences in the dependent variables of interest at post-testing. For each variable, the percentage of change was calculated compared with baseline.

This analysis approach (i.e. post-test performance as the outcome with baseline performance as a covariate) allowed us to compare post-testing outcomes, while accounting for potential baseline group differences. In the case of a significant omnibus test, pairwise comparisons were performed to examine potential between-group differences. These pairwise comparisons were based on the adjusted group means. In addition, 95% confidence intervals (CI95%) were calculated based on the adjusted group mean differences and Cohen’s d effect size (ES) statistics were calculated by dividing the adjusted group mean differences by the larger of the group standard deviations. The Bryant-Paulson procedure was used when conducting the pairwise comparisons and calculating the confidence intervals. An alpha of 0.05 was used for all significance tests. Effect sizes of 0.2, 0.5, and 0.8 were considered ‘small’, ‘moderate’, and ‘large’. SPSS software was used for statistical analysis (IBM Corp., Armonk, NY, USA).

### Quality assurance

Quality of data’s checked by authors.

### Expected outcomes of the study

This study suggests that adding a smartphone app to GPR could significantly relieve pain, and improve disability, endurance, forward head angle and quality of life in people with neck pain and FHP compared with GPR alone. Further research is needed to confirm the clinically improvement differences on symptoms in people with neck pain and FHP when adding a smartphone app to GPR.

### Dissemination of results and publication policy

Not applicable.

### Duration of the project

| **ecruitment status** | Completed |
| --- | --- |
| **Date of protocol fixation** | | 2018 | Year | 09 | Month | 11 | Day | | --- | --- | --- | --- | --- | --- | |
| **Date of IRB** | | 2018 | Year | 09 | Month | 20 | Day | | --- | --- | --- | --- | --- | --- | |
| **Anticipated trial start date** | | 2018 | Year | 10 | Month | 01 | Day | | --- | --- | --- | --- | --- | --- | |
| **Last follow-up date** | | 2019 | Year | 01 | Month | 01 | Day | | --- | --- | --- | --- | --- | --- | |
| **Date of closure to data entry** | | 2020 | Year | 01 | Month | 01 | Day | | --- | --- | --- | --- | --- | --- | |
| **Date trial data considered complete** | | 2020 | Year | 01 | Month | 01 | Day | | --- | --- | --- | --- | --- | --- | |
| **Date analysis concluded** | | 2020 | Year | 01 | Month | 01 | Day | | --- | --- | --- | --- | --- | --- | |

### Problems anticipated

Not applicable.

### Project management

Fatemeh Abadiyan (F.A), Malihe Hadadnezhad (M.H), Zohre Khosrokiani (Z.KH), Amir Letafatkar (A.L), Haniyeh Akhshik (H.A)

A.L., Z.KH., M.H., H.A, and F.A. prepared study design and implement a testing, A.L., and Z.KH. wrote the main manuscript text and M.H. and Z.KH. participate in final edition of article.

### Ethics

This study was performed in accordance with the 1964 Helsinki declaration, its later amendments and local ethics committee. This study was approved by an Kharazmi University Institutional Review Board in human subjects (DBSI12052019).

### Informed consent forms

Prior to participation at the study, all subjects were explained about the objectives and provided written informed consent and all participants provided written informed consent prior to enrollment.

#### Budget

None.

#### Other support for the project

None.

#### Collaboration with other scientists or research institutions:

#### None.

#### Links to other projects:

#### none.

#### Curriculum Vitae of investigators

Fatemeh Abadiyan currently works at the Department of Biomechanics and Sports Injuries, Kharazmi University. Their current project is 'Posture, and Pain'.

[Kharazmi University](https://www.researchgate.net/institution/Kharazmi_University)

Department of Biomechanics and Sport Injuries

She currently works at the Department of Biomechanics and Sport Injuries, University of Kharazmi. His expertise is in posture, pain, and injury prevention of athletic performance and mechanisms.

Malihe Hadadnezhad currently works at the Department of Biomechanics and Sports Injuries, Kharazmi University. Their current project is 'Posture, and Pain'.

[Kharazmi University](https://www.researchgate.net/institution/Kharazmi_University)

Department of Biomechanics and Sport Injuries

She currently works at the Department of Biomechanics and Sport Injuries, University of Kharazmi. His expertise is in posture, pain, and injury prevention of athletic performance and mechanisms.

Zohre Khosrokiani currently works at the Department of Biomechanics and Sports Injuries, Kharazmi University. Their current project is 'Posture, and Pain'.

[Kharazmi University](https://www.researchgate.net/institution/Kharazmi_University)

Department of Biomechanics and Sport Injuries

She currently works at the Department of Biomechanics and Sport Injuries, University of Kharazmi. His expertise is in posture, pain, and injury prevention of athletic performance and mechanisms.

Amir Letafatkar

Amir Letafatkar currently works at the Department of Biomechanics and Sports Injuries, Kharazmi University. Their current project is 'Posture, Pain and Injury'.

[Kharazmi University](https://www.researchgate.net/institution/Kharazmi_University)

Department of Biomechanics and Sport Injuries

Head of Department, Feb 2009 - Present

Amir Letafatkar currently works at the Department of Biomechanics and Sport Injuries, University of Kharazmi. His expertise is in posture, pain, and injury prevention of athletic performance and mechanisms.

Haniyeh Akhshik currently works at the Department of Biomechanics and Sports Injuries, Kharazmi University. Their current project is 'Posture, and Pain'.

[Kharazmi University](https://www.researchgate.net/institution/Kharazmi_University)

Department of Biomechanics and Sport Injuries

She currently works at the Department of Biomechanics and Sport Injuries, University of Kharazmi. His expertise is in posture, pain, and injury prevention of athletic performance and mechanisms.

#### Other research activities of the investigators

Note involved.

#### Financing and insurance

None.
