# Supplementary figures and images for: Adding a smartphone app to global postural re-education to improve neck pain, posture, quality of life, and endurance in people with nonspecific neck pain: a randomized controlled trial
Source: Trials. 2021 Apr 12;22:274. doi: 10.1186/s13063-021-05214-8 (PMC8042925; doi:10.1186/s13063-021-05214-8)

RCT Protocol


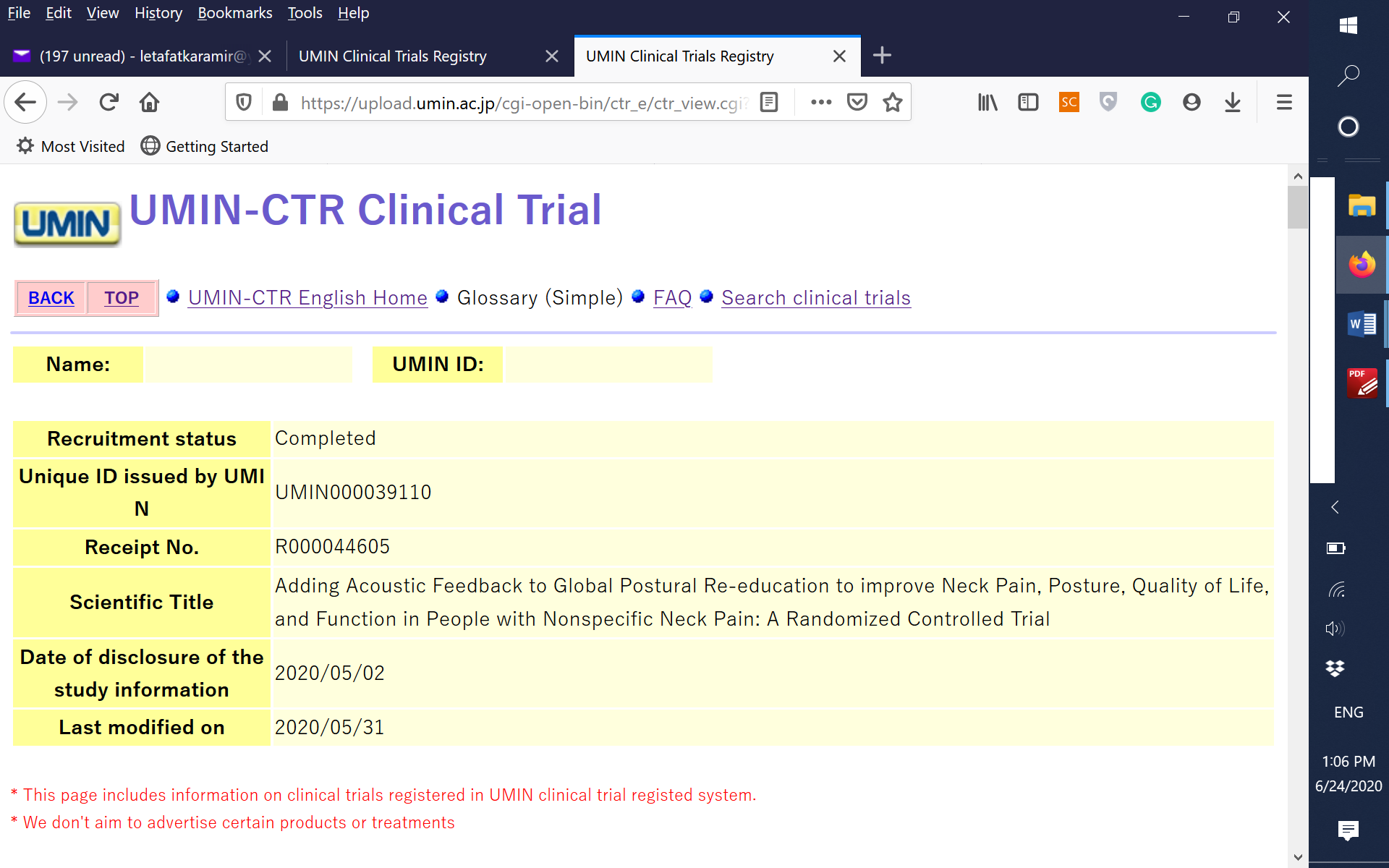


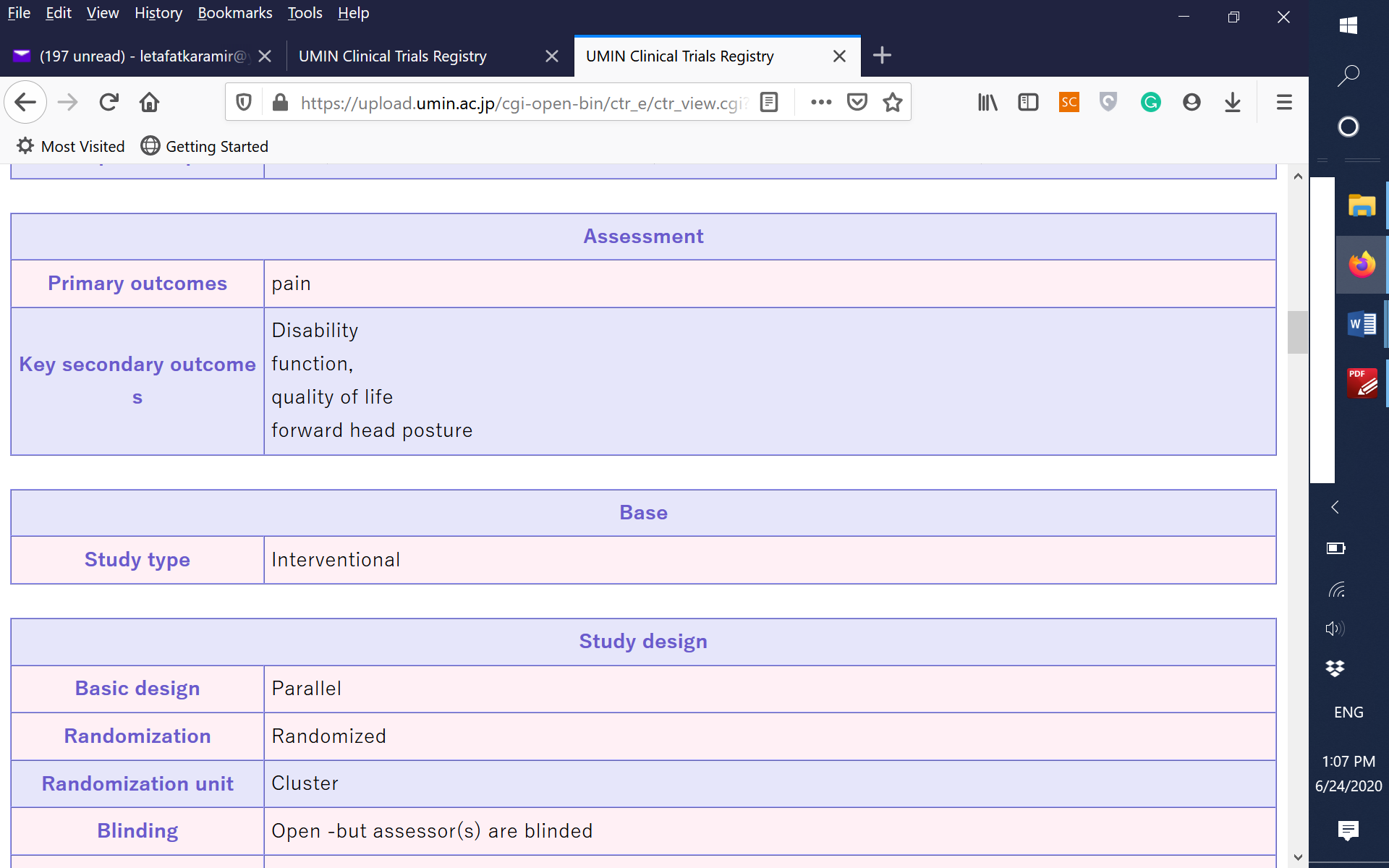


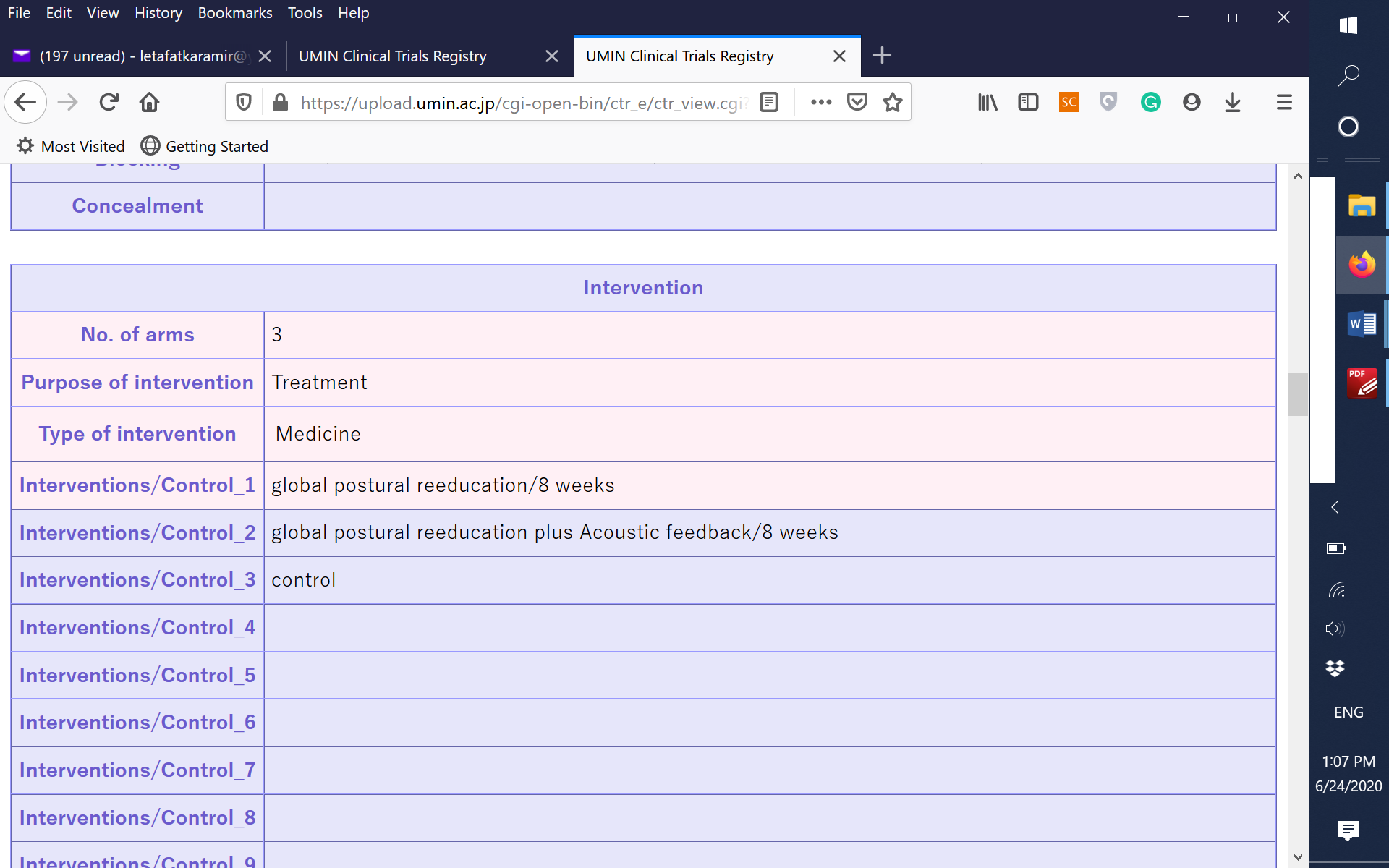


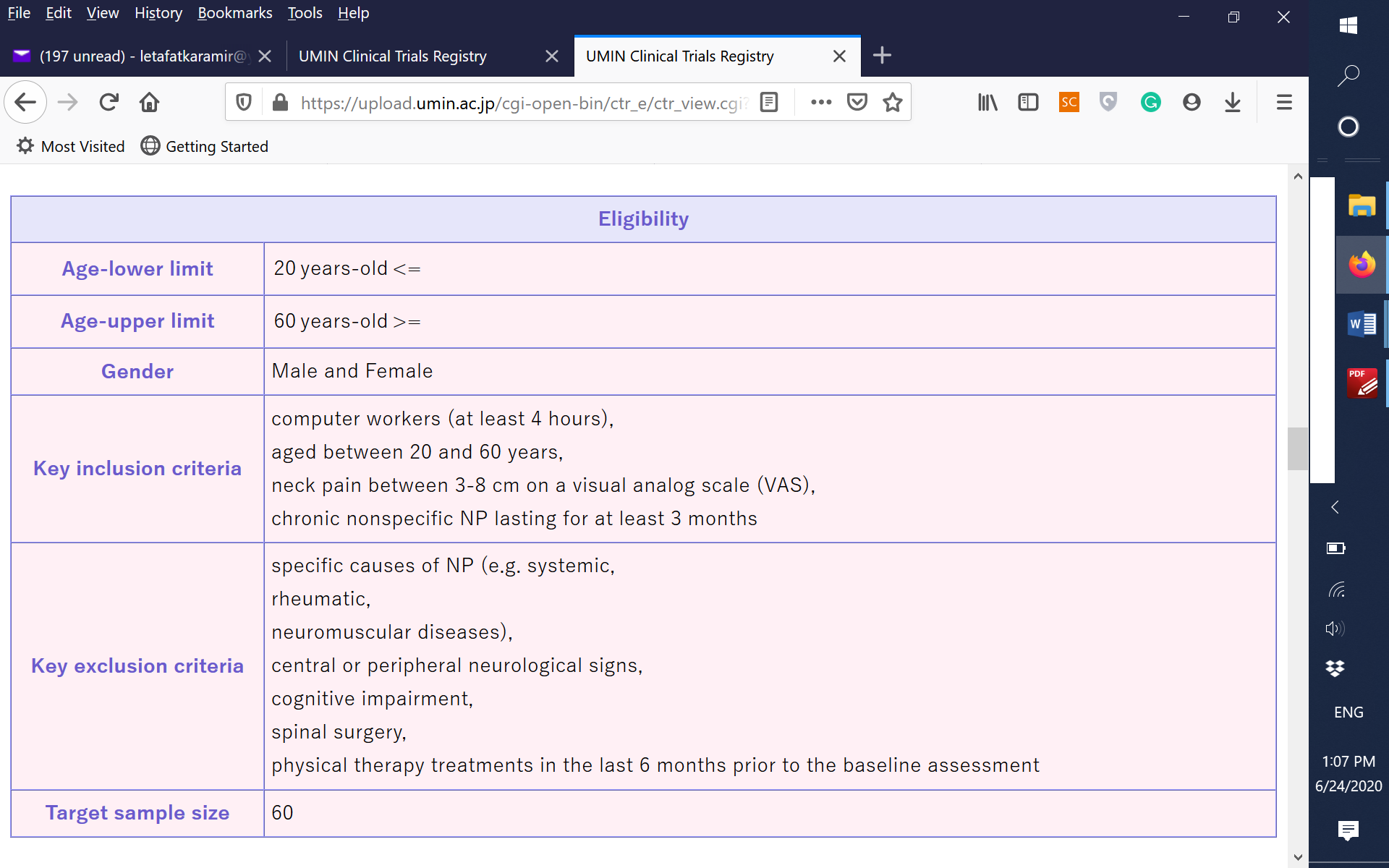


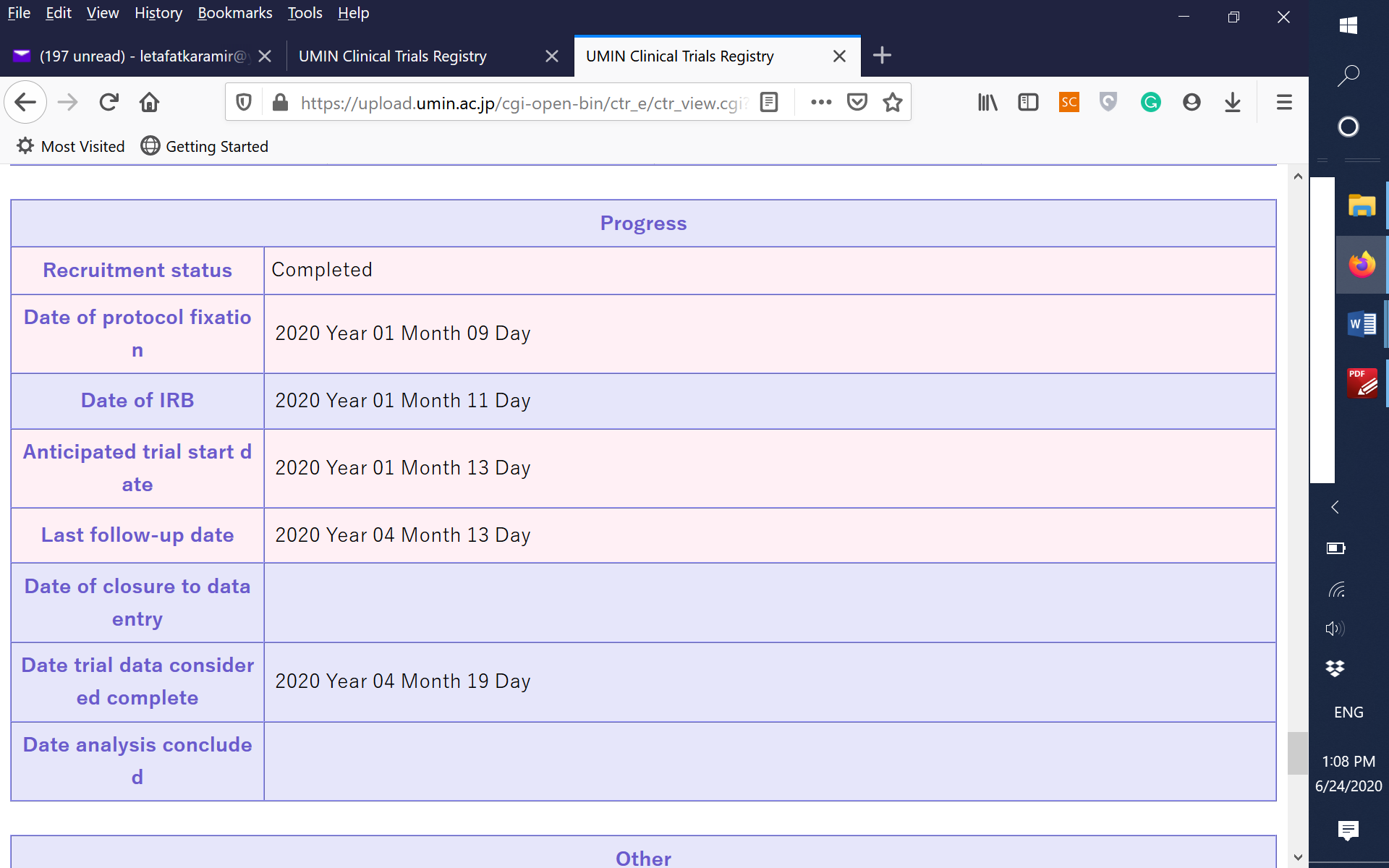


**Link to view the page**

https://upload.umin.ac.jp/cgi-open-bin/ctr_e/ctr_view.cgi?recptno=R000044605

Supplement: Supplementary file 2 — Additional file 2. RCT Protocol. [file 13063_2021_5214_MOESM2_ESM.doc]

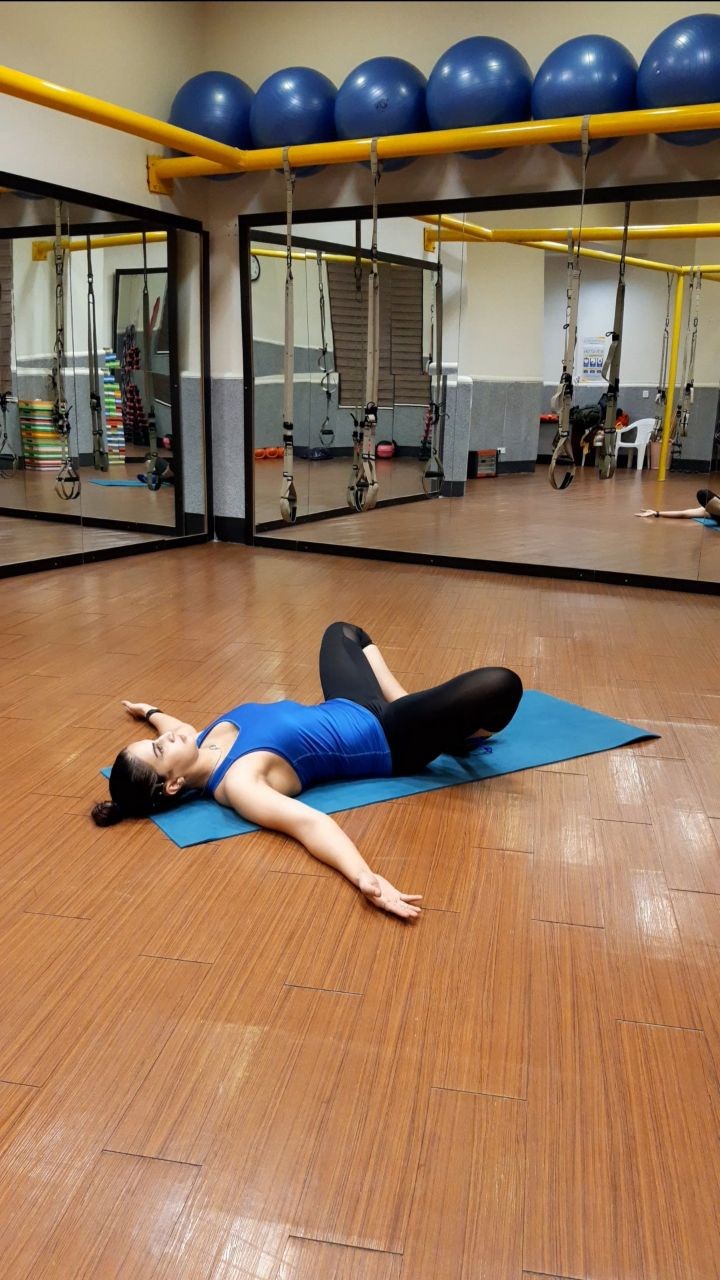

Supplement: Supplementary file 3 — Additional file 3. Exercise 1- Start. [file 13063_2021_5214_MOESM3_ESM.jpg]

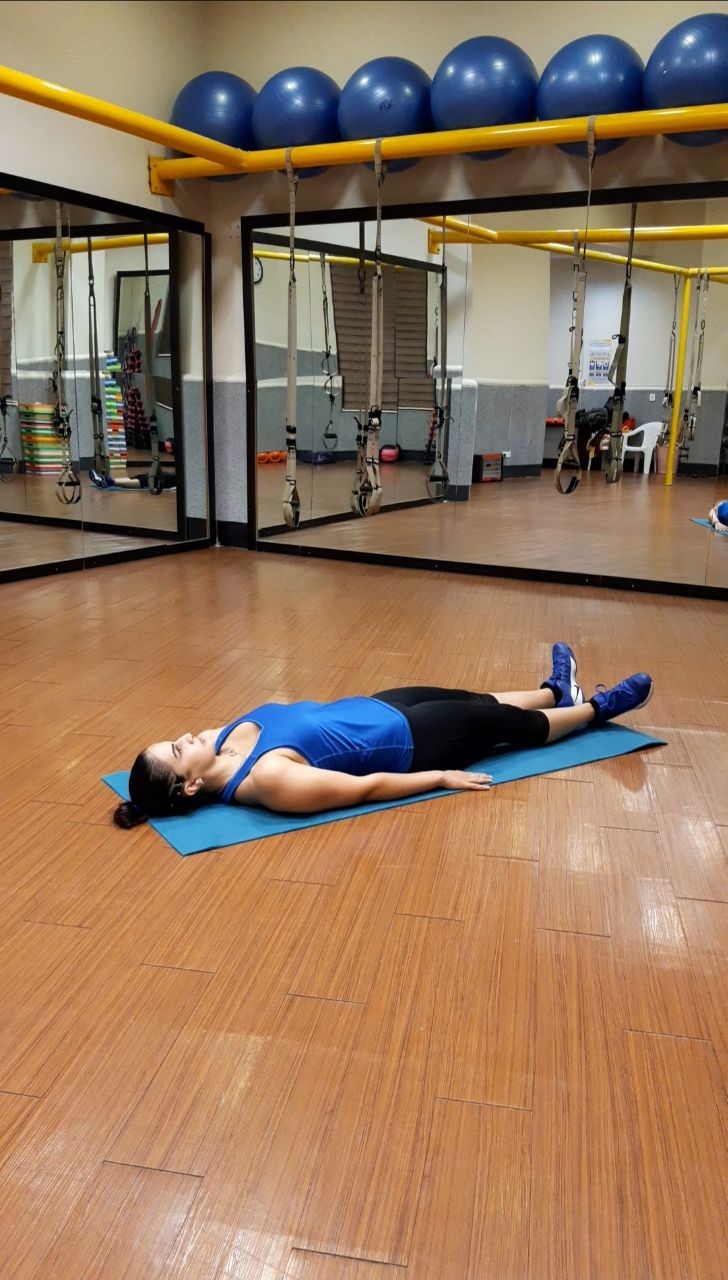

Supplement: Supplementary file 4 — Additional file 4. Exercise 1-End. [file 13063_2021_5214_MOESM4_ESM.jpg]

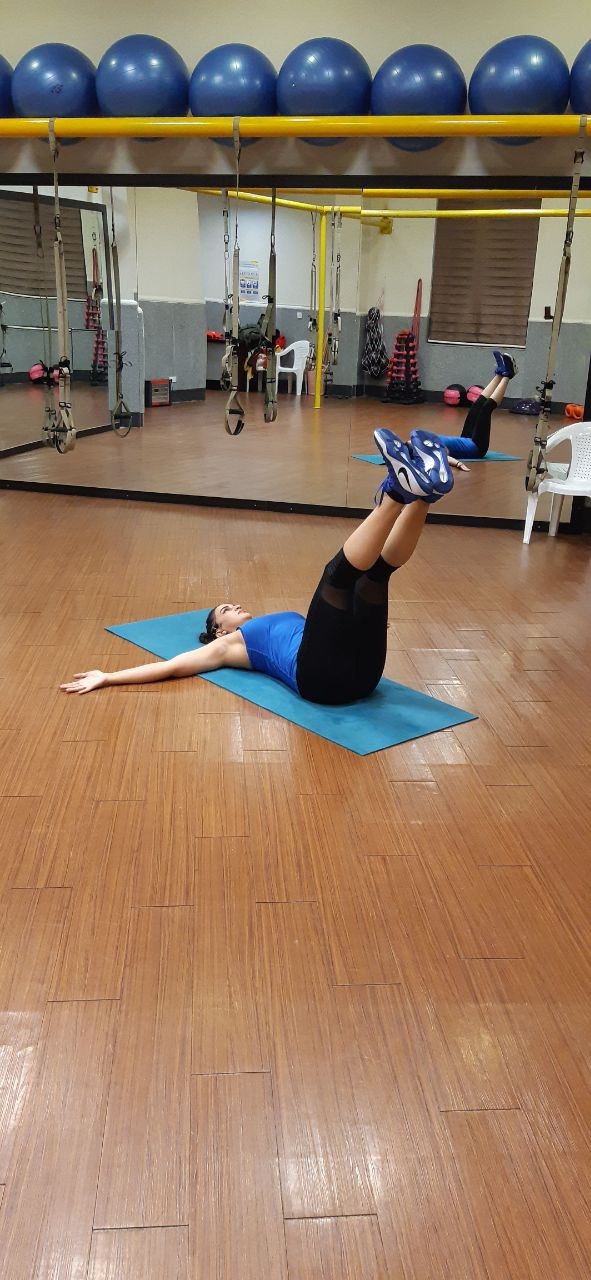

Supplement: Supplementary file 5 — Additional file 5. Exercise 2-Start. [file 13063_2021_5214_MOESM5_ESM.jpg]

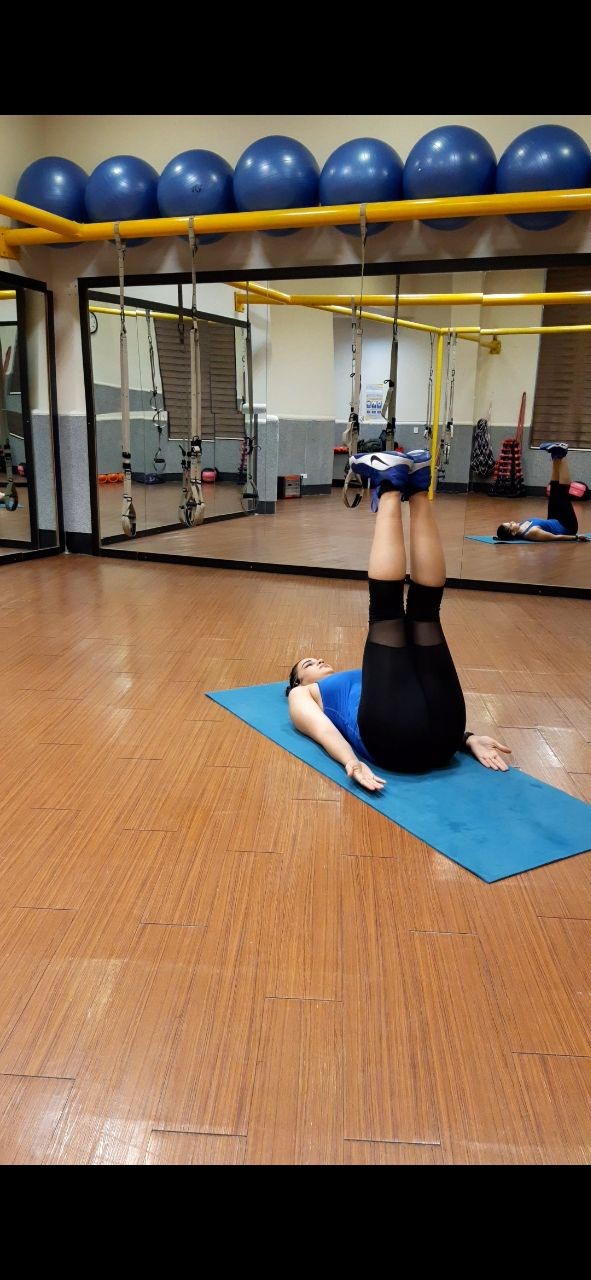

Supplement: Supplementary file 6 — Additional file 6. Exercise 2-End. [file 13063_2021_5214_MOESM6_ESM.jpg]
